# Functionalization of the Surface of Ti6Al4V Alloy Samples Printed Using Additive Technology DMLS for Orthopedic Applications Using Glow Discharge Treatment

**DOI:** 10.3390/ma19081604

**Published:** 2026-04-16

**Authors:** Gabriela Wielgus, Wojciech Kajzer, Julia Lisoń-Kubica, Aleksandra Żurawska, Jakub Wężowicz, Tomasz Borowski, Bogusława Adamczyk-Cieślak, Anita Kajzer

**Affiliations:** 1 Department of Biomaterials and Medical Devices Engineering, Faculty of Biomedical Engineering, Silesian University of Technology, Roosevelta 40 Street, 41-800 Zabrze, Poland; julia.lison-kubica@polsl.pl (J.L.-K.); az306892@student.polsl.pl (A.Ż.); jw301192@student.polsl.pl (J.W.); anita.kajzer@polsl.pl (A.K.); 2 Department of Biomechanics, Faculty of Biomedical Engineering, Silesian University of Technology, Roosevelta 40 Street, 41-800 Zabrze, Poland; wojciech.kajzer@polsl.pl; 3 Faculty of Materials Science and Engineering, Warsaw University of Technology, 02-507 Warsaw, Poland; tomasz.borowski@pw.edu.pl (T.B.); boguslawa.cieslak@pw.edu.pl (B.A.-C.)

**Keywords:** Ti64 ELI powder, additive technologies, nitrogen layers, personalized orthopedic implants, testing of mechanical and physicochemical properties

## Abstract

Previous studies of nitrogen and carbonitride layers on titanium alloys have mainly focused on cast or wrought materials. These traditional manufacturing methods are increasingly being replaced by additive methods, which allow the geometry of the manufactured product to be personalized. In the case of multi-component structures, and implant systems in particular, the hardness and abrasion resistance of the surface are insufficient. Therefore, these surfaces must be modified to improve these properties. Therefore, the aim of this work was to evaluate the properties of surface-modified Ti64 ELI alloy samples produced by the additive Direct Metal Laser Sintering method. To increase the hardness and abrasion resistance of the surface, a diffusion layer of TiN was produced under glow discharge conditions on samples previously heat-treated at temperatures of 800 °C, 910 °C, and 1020 °C. Since these implants remain in the body, it is important to sterilize them beforehand. Therefore, this study included samples after steam sterilization, and the results were compared to unsterilized samples. This study evaluated the structure of the material, the phase composition of the layer, the topography and wettability of the surface, along with the surface energy (before sterilization θ_av_ > 106°), resistance to pitting corrosion, hardness, and tribological properties.

## 1. Introduction

Metal biomaterials, particularly titanium, are the dominant materials used for implants in reconstructive and surgical procedures [1,2,3,4,5,6,7,8]. Metal biomaterials, particularly titanium, are the dominant materials used in implants for reconstructive and surgical procedures. Over the past few decades, they have undergone a remarkable evolution, transforming from an innovative material used in bone implants to one of the most used materials in orthopedics and dentistry. Initially, the biocompatibility and corrosion resistance of titanium made it the preferred choice over other metallic materials. Over time, technological advances have enabled the development of titanium alloys and surface modifications that have increased osteoconductivity, mechanical properties, and wider application of titanium-based implants. Recent advances in additive manufacturing and nanotechnology have further revolutionized the use of titanium, enabling the production of implants tailored to the individual needs of the patient [9]. The most complex issues arise when selecting the parameters for the manufacturing processes of surface layers on implants used for reconstruction in bone and joint and cardiovascular systems. Most of the research focuses on modifying the surface of these products manufactured using traditional methods, such as forging and rolling. The surface of these products should be characterized by adequate biocompatibility to ensure resistance to corrosion: pitting, crevice, stress, fatigue, or tribological characteristics. Degradation products that occur in multi-element structures should be inert to surrounding tissues.

Due to its favorable physicochemical properties and susceptibility to deformation, the Ti6Al4V alloy [10] remains the most used material to produce orthopedic implants. It is characterized by high availability, corrosion resistance, and good biocompatibility. Orthopedic implants are increasingly being customized to achieve the best possible stabilization results. For this purpose, an additive technology is used, which is an innovative method for manufacturing personalized implants from metal powders. Methods such as SLM (Selective Laser Melting), DMLS (Direct Metal Laser Sintering), and PBF (Powder Bed Fusion) are used [11,12,13,14,15,16]. Such implants are increasingly used to treat osteoarthritis, which is a serious health problem affecting an increasing number of people, especially the elderly. It results from progressive degenerative changes in the articular cartilage, leading to pain and impaired biomechanics of movement. These processes are often associated with mechanical overload and age-related changes, affecting patients’ daily lives and making it difficult for them to perform basic activities [17]. To improve tribological properties and biomechanics of movement, implant surfaces are subjected to mechanical polishing and chemical etching, which allows for the removal of particles remaining after the powder printing process and reduces friction between components [18]. After surface treatment, the final surface roughness (Ra) is 3.69 ± 0.10 µm, which is particularly important for small implants, such as phalangeal implants. Nevertheless, during fatigue testing, material wear and the presence of incompletely melted Ti6Al4V alloy powder particles were observed, which is a negative phenomenon on the implant surface. The pattern of scratches distributed uniformly on the contact surfaces was similar on all prostheses and indicated wear on the mating surfaces. This correlates with the wear of metal–metal joints. This type of damage can lead to faster wear of implants and the migration of fine metal powder particles, causing joint blockage and the release of Al and V ions into the surrounding tissues. In particular, the presence of vanadium in the powder can cause inflammatory reactions in the body. It is a toxic element with neurogenic effects that can induce inflammation and osteolysis [19,20], which increases the risk of revision surgery. On the other hand, Al aggregation in tissues is correlated with the occurrence of, among other health conditions, Alzheimer’s disease [21]. Therefore, it is necessary to modify their surface in order to improve the biocompatibility and functionality of the implants produced, because even when a non-toxic material is used, the implant is still a foreign body to the organism, which may be rejected if the response is inadequate.

The surface modification of implants made of titanium alloys is most often carried out using an anodic oxidation process, which allows the creation of oxide layers with varying thickness and morphology and favorable physicochemical properties, especially those with a dominant share of TiO_2_ [22,23,24]. In this as well as the last century, many technologies for modifying the surface of metal implants have been introduced. These include, among others, surface layers of the TiN + Ti_2_N + αTi(N) and Ti(CN) + Ti_2_N + αTi(N) type, produced after nitriding and nitrocarburizing on titanium and its alloys, with a specific microstructure, phase and chemical composition, as well as surface topography. X-ray analysis allows the identification of the phase composition of the layer and the determination of residual stresses in the produced layer [25,26,27]. Residual stresses have a direct impact on the properties of the titanium alloy on which the layer was produced. Analysis of diffractograms in terms of residual stress for diffusion layers indicates there is a dependence on the type of nitriding process used. In the case of phase analysis, the shift in αTi-phase peaks indicates the formation of an αTi(N) diffusion zone, directly above which are the other nitriding products TiN and Ti_2_N [28,29,30]. Glow discharge processes make it possible to create diffusion-type surface layers with an outer zone of nanocrystalline TiN or Ti(CN) [31,32,33], which should guarantee greater hardness and resistance to wear by friction, which is not demonstrated by artificially thickened ceramic TiO_2_ oxide layers. The passive layer formed on the surface of diffusion layers often exhibits more favorable biological properties than the alloy without the layer [34,35]. The processes of nitriding and ion nitrocarburizing presented in the world literature mainly concern the Ti6Al4V alloy, which is cast and plastically processed [36]. At the same time, there is a lack of data on the use of plasma techniques for the functionalization of the surfaces of Ti6Al4V alloy implants produced using additive methods. The surface engineering techniques used for titanium alloys produced by additive methods mainly include oxidation methods [37,38] or magnetron sputtering [39]. Therefore, the primary objective of this study was to evaluate the properties of surface-modified Ti6Al4V ELI alloy specimens produced by the DMLS method and intended for orthopedic implants, with particular emphasis placed on the mechanical and physicochemical properties of the substrate and the diffusion nitride layer, considering the steam sterilization process.

## 2. Materials and Methods

Samples with a diameter of 14 mm and a thickness of 3 mm, made of Ti64ELI alloy powder with a particle diameter of 39 ± 3 μm using the DMLS method on an EOS M100 printer (EOS GmbH, Krailling, Germany), were selected for testing. The chemical composition of the powder was consistent with the EOS Titanium Ti64 Grade 23 material data sheet [40,41]. Next, to remove unmelted powder, the samples were sandblasted with glass beads with a diameter of 90 μm to 150 μm in a Renfert “VARIObasic” sandblaster at a pressure of 6 bar (Renfert GmbH, Hilzingen, Germany). The process lasted 120 s at an angle of 45°.

**Figure 1:**
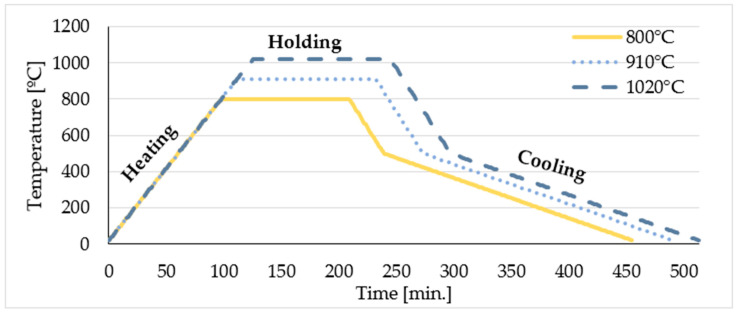
Stages of heat treatment.

The samples were then divided into four groups, which were heat-treated in an AMAZEMET inFURNER vacuum furnace (Amazemet Spółka z ograniczoną odpowiedzialnością, Warsaw, Poland) (Figure 1) with the following process parameters:

- **Group I**: As-built samples—samples in their initial state.
- **Group II**: The specimens were heat-treated in a vacuum furnace at a temperature of 800 ± 10 °C for 2 h, followed by rapid cooling in an expanding argon atmosphere to 500 °C. In the final stage, the samples were cooled together with the furnace to room temperature.
- **Group III**: The specimens were heat-treated in a vacuum furnace at a temperature of 910 ± 10 °C for 2 h, followed by rapid cooling in an expanding argon atmosphere to 500 °C. In the final stage, the samples were cooled together with the furnace to room temperature.
- **Group IV**: The specimens were heat-treated in a vacuum furnace at a temperature of 1020 ± 10 °C for 2 h, followed by rapid cooling in an expanding argon atmosphere to 500 °C. In the final stage, the samples were cooled together with the furnace to room temperature.

In the next stage, the samples were pre-ground on a Struers Tegramin-30 grinding and polishing machine (Struers ApS, Ballerup, Danmark), cleaned in a Struers Lavamin ultrasonic cleaner, and then embedded in Purifast polypropylene resin using a Struers CitoPress-30 automatic press (Struers ApS, Ballerup, Danmark) at a temperature of 150 °C and a pressure of 250 bar for 3 min. The final stage consisted of mechanical surface treatment. The sections were ground on P320, P500, P800, P1200, and P2000 grit sandpaper and mechanically polished on a polishing wheel using silicon oxide. On the prepared samples, a diffusion nitride layer was produced under glow discharge conditions using a titanium active screen with the following process parameters: temperature T = 750 °C; current—7 A; voltage—985 V; time—8 h; pressure—2 mbar; and gas atmosphere composition: N_2_—0.95 mL/min and H_2_—5 mL/min. Additionally, part of the samples was sterilized with steam in a Mocom Basic Plus autoclave at a temperature of T = 134 °C and pressure of 2.10 bar for 12 min (Mocom Srl, Imola, Italy). The samples were then divided according to Table 1.

### 2.1. Material Structure

Optical metallographic examinations were carried out using a Leica DMi8 optical microscope at magnifications of 200× and 1000× (Leica Microsystems GmbH, Wetzlar, Germany). For this purpose, polished metallographic samples were prepared. In the first stage, specimens from each analyzed group were mounted in PolyFast resin at a temperature of 180 °C and a pressure of 250 bar. Subsequently, mechanical surface preparation was performed. The sections were ground on abrasive papers with grit sizes of P320, P500, P800, P1200, and P2000. In the next stage, polishing was carried out on polishing cloths using colloidal SiO_2_ suspension. The final step involved etching the surface in a solution consisting of 10 mL of hydrofluoric acid and 30 mL of distilled water for 15 s.

### 2.2. XRD Layer Structure Analysis

Diffraction tests and phase analysis were performed on four groups of samples. Diffraction records were made on a Bruker D8 ADVANCE X-ray diffractometer (Bruker AXS GmbH, Karlsruhe, Germany) using Cu Ka radiation (λ = 0.154056 nm) at room temperature. The recording conditions were as follows: voltage—40 kV; current—40 mA; angular range 2θ—from 15° to 100°; step D2θ—0.025°; counting time—5 s; fixed angle of incidence θ = 1°. Based on the diffraction records, a phase analysis of the tested samples was performed by matching the crystal phase patterns to the obtained diffraction records using the PDF-2 Release 2025 database available in the laboratory.

### 2.3. Surface Roughness

The topography of the selected samples was analyzed using a Leica Microsystem optical profilometer (Leica Microsystems GmbH, Wetzlar, Germany); based on this, the average surface roughness value Sa [μm] was determined [42]. Three measurements were performed at room temperature on samples from each temperature group.

### 2.4. Wettability and Surface Energy

The wettability and surface energy tests were performed using the sessile drop method with a Biolin Scientific Attension Theta Flex goniometer (Biolin Scientific, Gothenburg, Sweden). Both distilled water and diiodomethane were used for the measurements. Two samples from each temperature group were used for the test. Five drops of distilled water and diiodomethane, each with a volume of 1.5 μL, were applied to the surface of the samples. Contact angle measurements were performed at a sampling frequency of 1 Hz at room temperature, T = 22 ± 1 °C. To determine the surface free energy, the Owens–Wendt method and the Biolin Scientifien computer program were used, which allows this value to be read automatically by comparing the contact angles for both liquids (θ_w_ and θ_d_). Values of the surface free energy (SFE) and their components, polar and dispersion, are as follows:

- For distilled water, the polar component $\gamma_{s}^{p}$ is 51.0 mJ/m^2^, while the dispersion component $\gamma_{s}^{d}$ is 21.8 mJ/m^2^,
- In the case of diiodomethane, the polar component $\gamma_{s}^{p}$ is 6.7 mJ/m^2^, and the dispersion component $\gamma_{s}^{d}$ is 44.1 mJ/m^2^.

### 2.5. Pitting Corrosion Resistance Testing

The corrosion resistance test was performed using the potentiodynamic method on a VoltaLab PGP 201 potentiostat in accordance with ISO 10993-15 [43] at room temperature, T = 22 ± 1 °C, and 250 mL of Phosphate-Buffered Saline (PBS) with a pH of 7.4. The pitting corrosion resistance tests were carried out on three samples from each subgroup.

The test was performed using an electrochemical cell in which the following electrodes were immersed: the reference electrode—Ag/AgCl (3 M KCl); auxiliary platinum electrode; and working electrode—the tested sample. First, the opening potential (E_ocp_) was determined under no-current conditions for 15 min, and then the initiation potential E_ini_ = E_ocp_—100 mV was calculated. The potential changed in the anodic direction at a rate of 3 mV/s, and the measurement surface area was 0.8 cm^2^. Based on the obtained graphs and the Stern method, the following parameters were determined: corrosion potential E_corr_ [mV], breakdown potential E_np_ [V], repassivation potential E_cp_ [V], and polarization resistance R_p_ [kΩ∙cm^2^].

### 2.6. Macroscopic Observations

The surface was evaluated both before and after the pitting corrosion resistance test using a Leica DMi8 digital microscope at 180× magnification.

### 2.7. Hardness

The hardness of the samples was measured using the Vickers method in accordance with ISO 6507-1 [44] on a Struers DuraScan hardness tester. A diamond pyramid penetrator with a square base and a vertex angle of 136° was used. To determine the surface hardness profile, the diamond indenter was pressed into the sample surface six times using different load values, respectively: HV0.3, HV0.5, HV1, HV3, HV5, HV10. Three samples from each temperature group were selected for testing (and the measurement was carried out five times for each sample), and the results were generated in a computer program; they were then compared and presented in graphical form.

### 2.8. Tribological Testing

#### Abrasion Wear Resistance Test

The abrasion resistance of the coating was tested using the ball-on-disc method with an Anton Paar TRB tribometer (Anton Paar GmbH, Graz, Austria), applying a load of 1 N. A ball made of AISI 440-C stainless steel (Ra ≤ 0.05 µm) with a diameter of 6 mm was used as a counter sample in the friction pair with the tested material, and the table with the tested material rotated at a speed of 2 cm/s. The measurements were carried out by setting the distance traveled by the ball to 24 m. The resistance to movement was determined during technically dry friction—TDF (Test of Dry Friction). All tribological tests were repeated three times with the parameters specified in Table 2.

Next, after conducting tribological tests to assess surface morphology and analyze the geometric structure of the surface of the friction mark obtained, a Leica DCM8 optical profilometer with interferometric mode (with LeicaSCAN 6.6 software) was used at 20× magnification (Leica Microsystems GmbH, Wetzlar, Germany). A single measurement covered an area of 840 × 630 µm. At the same time, the wear area and wear depth were assessed to qualitatively determine the material’s resistance to abrasive wear.

### 2.9. Statistical Analysis

Assumptions of the normality of the distribution (Shapiro–Wilk test) and equality of variance were made. In all cases, these assumptions were met. Therefore, ANOVA was performed with *p* = 0.05. Post hoc analysis was performed using Tukey’s test.

## 3. Results and Discussion

### 3.1. Material Structure—Results and Discussion

The microstructure of the samples investigated is shown in Table 3.

A two-phase α + β structure was observed. The microstructure of the alloy after heat treatment at 1020 °C shows similarities to both the microstructure of the material in its initial state and after heat treatment at 910 °C. However, samples from the AB_I group are characterized by a predominance of the α-phase (light areas). In contrast, in heat-treated samples, a clear dominance of the β-phase is observed.

In the case of samples from the HT_II group, the structure consists of an α-phase matrix (grey areas) and an interlamellar β-phase (light areas), which results from the different etching rates of the individual phases, as confirmed by the literature data [45,46]. This effect is a consequence of differences in the chemical composition of individual phases, which translate into their diverse mechanical and physicochemical properties. In the case of heat treatment at higher temperatures, such as 910 °C and 1020 °C, an increased proportion of the β-phase in the structure is observed. However, rapid cooling leads to the transformation of the β-phase into a metastable α’-phase, which significantly affects the mechanical properties of the material, particularly its hardness.

### 3.2. XRD Layer Structure Analysis—Results and Discussion

**Figure 2:**
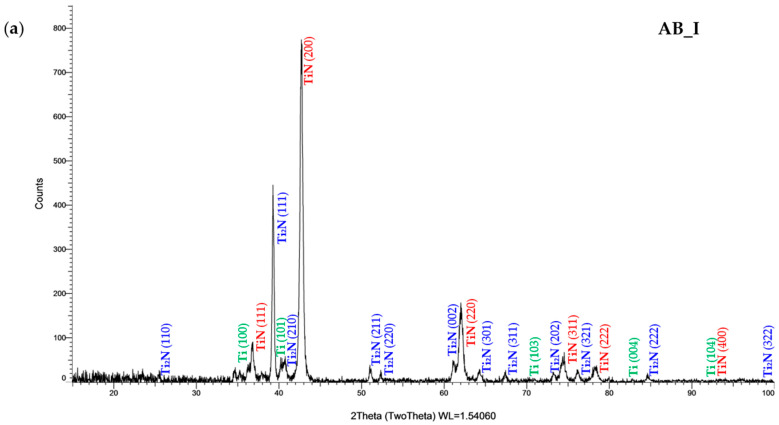

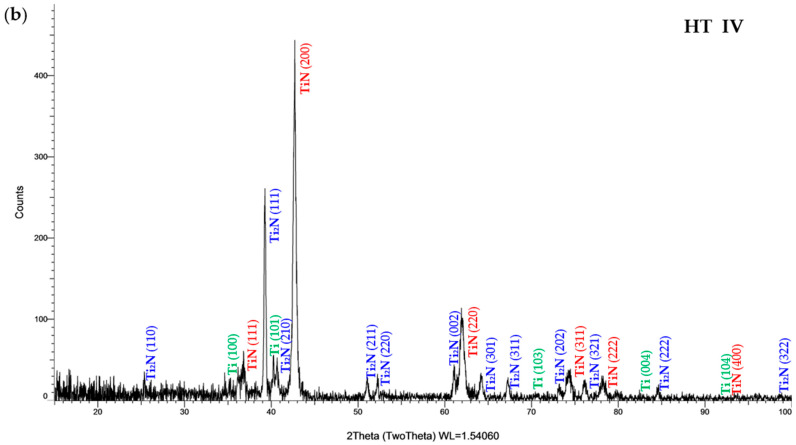
X-ray diffraction (XRD) pattern of samples (**a**) AB_I and (**b**) HT_IV.

The results of the layered structure are presented in Figure 2.

Based on the phase analysis, it can be concluded that the tested sample surfaces do not differ in phase composition or in peak intensities. This shows that the heat treatment of the alloy at 1020 °C had no effect on the structure of the layer. In all cases, the presence of TiN and Ti_2_N phases was observed, as well as diffraction peaks of low intensity originating from the titanium substrate. The TiN phase exhibited multiple peaks of varying intensity, with the highest intensity observed for the (200) plane. Peaks corresponding to the Ti_2_N phase were also detected at several angles, with the (111) plane showing the strongest intensity. According to previous studies on the nitriding of titanium alloys, the layer consists of a top, fine crystalline layer of TiN [47], followed by Ti_2_N [48].

### 3.3. Surface Roughness—Results and Discussion

**Figure 3:**
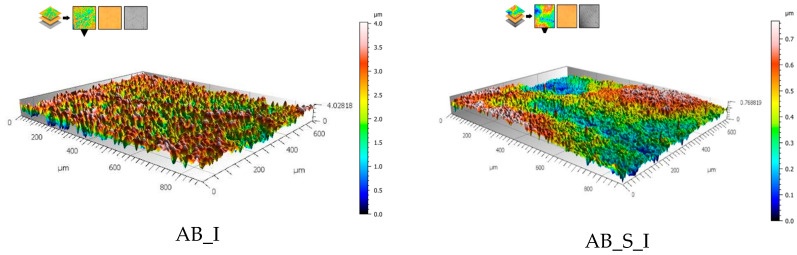

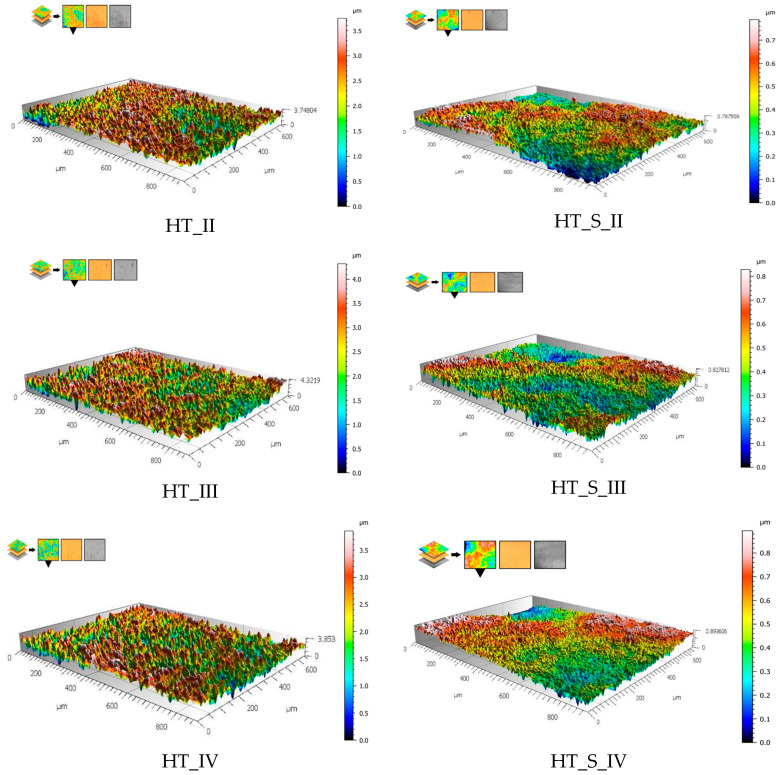
Topography maps of samples before and after sterilization.

Examples of surface topography maps of the samples are shown in Figure 3. A comparison of the Sa parameter for sample surfaces is presented in Table 4.

The creation of a diffusion nitrogen layer resulted in a significant increase in the Sa parameter value compared to the mechanically polished surface [13], for which the Sa parameter value ranged from 0.08 μm to 0.09 μm. In the case of the diffusion layer, values ranging from 0.52 μm to 0.62 μm were obtained. This effect is beneficial because the greater the surface area of the biomaterial, the better the osseointegration of the implant with the bone tissue. Furthermore, based on the results of surface roughness tests, a significant effect of steam sterilization on the surface area of the samples was observed (*p* < 0.05). Sterilization reduced the roughness of the surface for all tested groups, which also affected the wettability of the surface. Smaller differences in values were also obtained for samples after sterilization, for which the Sa parameter ranged from 0.11 μm to 0.13 μm.

### 3.4. Wettability and Surface Energy—Results and Discussion

**Figure 4:**
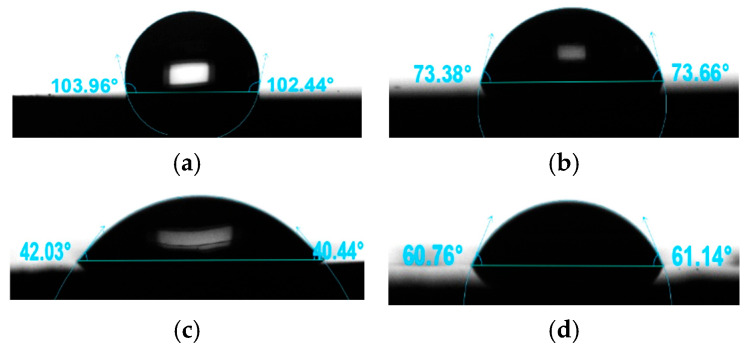
Determination of contact angles: (**a**) HT_III by distilled water, (**b**) HT_III by diiodomethane, (**c**) HT_S_III by distilled water, and (**d**) HT_S_III by diiodomethane.

Examples of droplets obtained during contact angle determination are shown in Figure 4.

**Figure 5:**
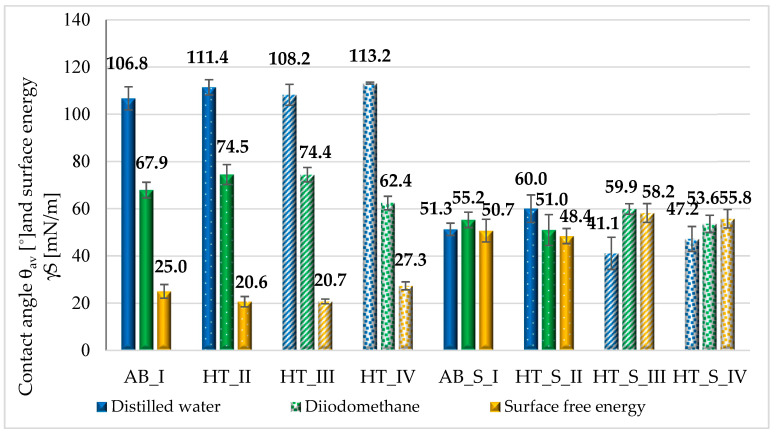
Contact angle and surface energy values of the tested samples.

The average values obtained for the contact angle with distilled water and diiodomethane, and the average surface energy values determined, are shown in Figure 5.

The surfaces of the samples before sterilization were hydrophobic, with a contact angle θ_av_ > 106°. Higher roughness values were obtained for these surfaces. However, the use of steam sterilization resulted in increased surface wettability and an increase in surface energy γ^S^ compared to the samples before sterilization (*p* < 0.05). The surfaces of the samples from the HT_S_III group were characterized by the highest wettability. This promotes biological activity, including cell proliferation processes on the surface, which is an important aspect in the context of orthopedic implant applications. The surface layer of the material should have appropriate adsorption properties towards proteins and support their biological activity, including the cell proliferation processes [47,49].

### 3.5. Pitting Corrosion Resistance Testing—Results and Discussion

**Figure 6:**
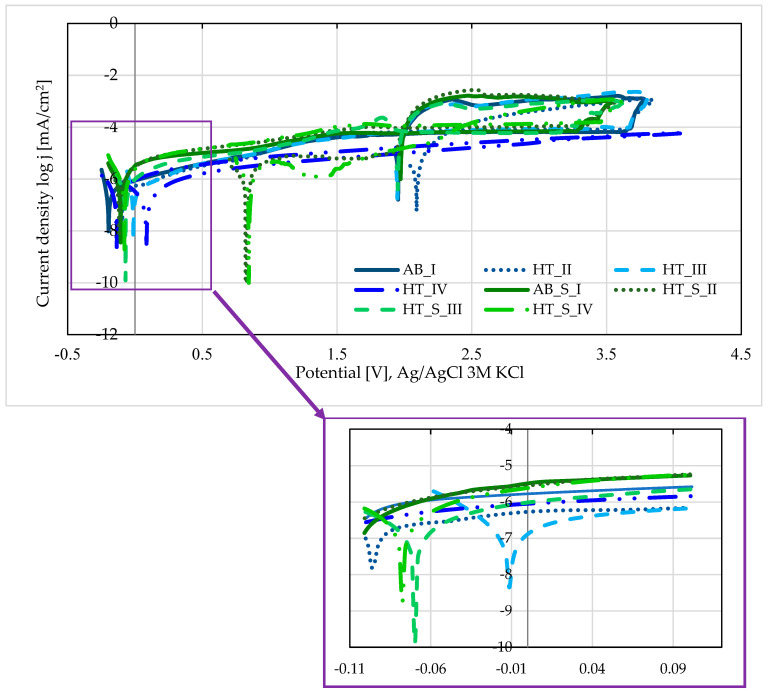
Polarization curves in logarithmic form for samples from two groups.

The polarization curves for two groups of samples are presented in Figure 6. The results indicate significant differences in pitting corrosion resistance between the sample groups investigated, as presented in Table 5. Hysteresis loops were observed in the graphs of all sample groups analyzed, which may indicate the occurrence of pitting corrosion. However, due to the high breakdown potential obtained (E_np_ > 3.2 V), it is concluded that the corrosion resistance is sufficient for implants made of this alloy that remain in the human body. According to ISO 10993-15 [43], a material is considered resistant to this type of corrosion if the breakdown or repassivation potential is above 2 V.

However, considering the polarization resistance values R_p_, a decrease in corrosion resistance can be observed for samples after steam sterilization (*p* > 0.05). On the other hand, for samples both before and after sterilization, the most favorable parameter values were obtained for group III, i.e., samples after heat treatment at 910 °C. The corrosion potential E_corr_ for samples from group HT_III before steam sterilization was −0.012 V. After steam sterilization (HT_S_III), this value was −0.062 V, which, compared to the other samples after steam sterilization, indicates the most favorable resistance to pitting corrosion. In the case of samples subjected to mechanical polishing without a layer, the polarization resistance value for samples after heat treatment at 910 °C was 63 kΩ·cm^2^ [13]. A similar value was recorded for samples from the HT_S_III group. The authors of publications [28] observed an increase in pitting corrosion resistance in samples made of the Ti6Al7Nb alloy, on which a diffusion layer with a TiN + Ti_2_N + αTi(N) structure was created because of the nitriding process. The corrosion potential of the Ti6Al7Nb samples was E_corr_ = −155 mV, while for samples with cathode potential (TiN-CP), the corrosion potential was 50 mV, and at plasma potential (TiN-PP), E_corr_ = −5 mV. The results obtained indicate that nitriding the surface of the Ti6Al7Nb alloy significantly affects the physicochemical properties of the material, improving its resistance to pitting corrosion.

### 3.6. Macroscopic Observations—Results and Discussion

To confirm the results of the pitting corrosion resistance test, macroscopic observations of the surface of the samples were carried out, as shown in Table 6.

The presence of hysteresis loops is confirmed by corrosion pits on the surfaces of samples from groups AB_I, HT_II, AB_S_I, and HT_S_II. No pitting corrosion was observed on the surfaces of samples showing greater resistance to pitting corrosion, which confirms the results of the tests presented in Section 3.5.

### 3.7. Hardness—Results and Discussion

**Figure 7:**
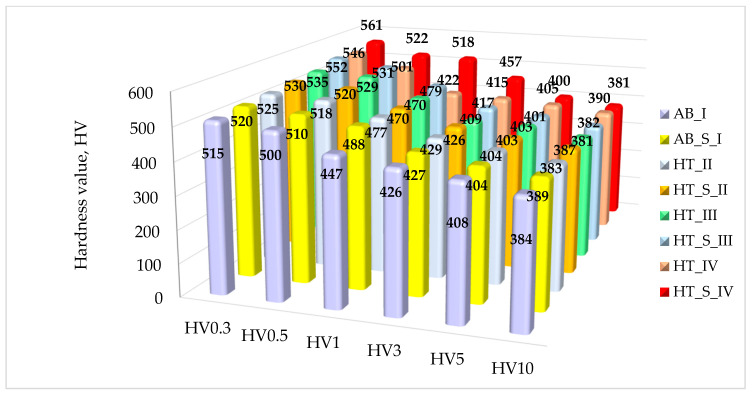
Hardness measurement results for all sample groups.

The relationship between the heat treatment temperature of the samples and their hardness under various loads is shown in Figure 7.

The observed hardness distribution confirms the presence of a TiN diffusion layer on the Ti64 ELI alloy substrate. The formation of the layer resulted in an increase in surface hardness, which decreased towards the metal substrate. Considering the purpose for which the diffusion layer was created, it can be concluded that the substrate hardness value was only obtained at a load of HV10, which was confirmed in an earlier publication by the authors [12,13]. The hardness values for the tested samples with a mechanically polished surface ranged from 349 to 390 HV5, depending on the heat treatment temperature used, which is related to the change in dislocation density and grain boundary distribution [48,50]. It was also found that sterilization caused a significant increase in hardness for all tested groups (*p* < 0.05).

### 3.8. Tribological Testing—Results and Discussion

#### Abrasion Wear Resistance Test—Results and Discussion

**Figure 8:**
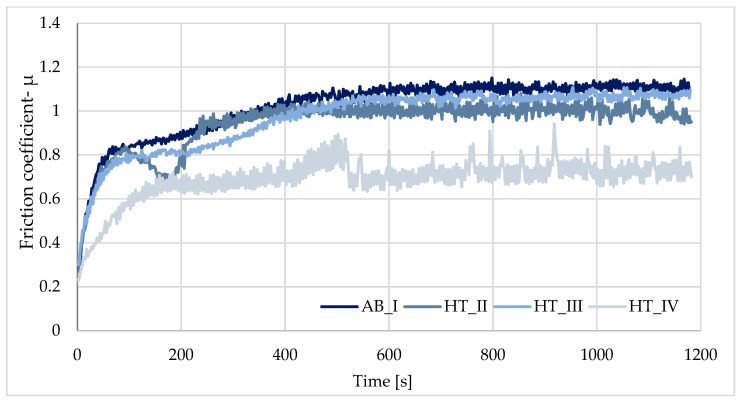
Examples of tribological test procedures for as-built + heat treatment samples.

**Figure 9:**
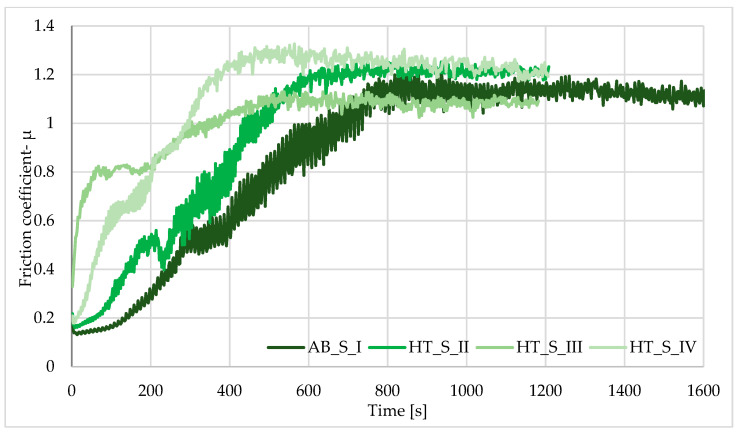
Examples of tribological test procedures for as-built samples after sterilization + heat treatment.

The highest average dry friction coefficient (TDF) value was observed for the AB_I sample from the first group and the HT_S_IV sample from the second group. A decrease in the same value was observed for the samples after heat treatment (HT_II, HT_III and HT_S_II, HT_S_III) before and after sterilization of the samples compared to the initial sample (*p* > 0.05). This means that the abrasion resistance after heat treatment and the sterilization process improved compared to the initial state. Tribological testing of the HT_S_IV samples showed a relatively high friction coefficient of f = 1.14, compared to the AB sample, where the friction coefficient was f = 1.00 (Figure 8 and Figure 9). The application of a sterilization process causes decreases in the friction coefficient—the results are shown in Table 7.

**Figure 10:**
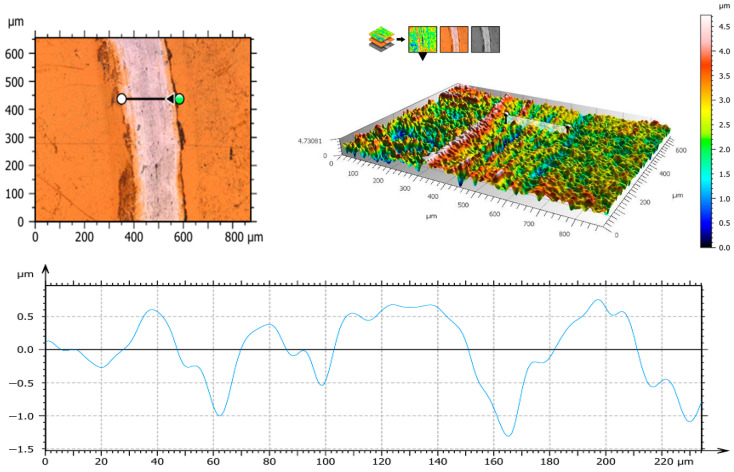
Example of isometric images of wear marks and wear profiles on a cross-section during dry friction TDF for samples.

**Figure 11:**
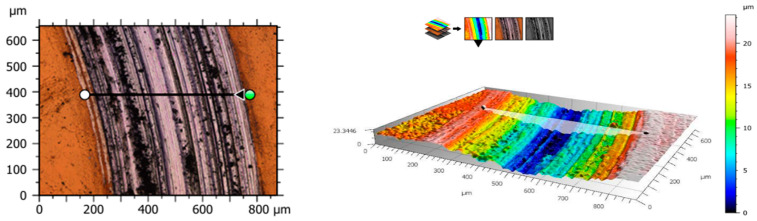

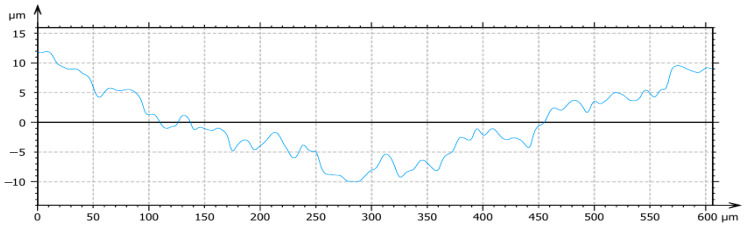
Example of isometric images of wear marks and wear profiles on a cross-section during dry friction TDF for samples after sterilization.

The most favorable friction coefficient value was observed for samples from the HT_S_III group. For these samples, the lower friction coefficient value indicates increased resistance of the produced layer to tribological wear. Image channels, based on which wear profiles and topographic maps of traces obtained after tribological tests were extracted, enabled observation of surface changes, as shown in Figure 10 and Figure 11. The results are indicated for the qualification assessment of abrasion value.

## 4. Conclusions

Titanium alloys, particularly Ti6Al4V, are the most commonly used biomaterials to produce customized orthopedic implants. However, a persistent problem in orthopedic implants is the wear of, for example, PE (polyethylene) inserts in the friction pair, which enables movement of the upper limbs. In addition, in the case of small orthopedic implants, such as phalangeal implants, where there is direct contact between metal elements, scratches and microdamage are observed on the friction surfaces. This results in the need for arthroplasty revision. Therefore, the aim of this study was to increase the hardness and resistance of Ti64ELI alloy samples obtained in the additive manufacturing process to tribological wear, which is crucial for the durability and functionality of the implant, while maintaining corrosion resistance. For this purpose, a diffusion layer with a TiN + Ti_2_N + αTi(N) structure was produced. The innovation of this approach stems from the fact that, according to the literature, nitriding processes mainly concern the Ti6Al4V alloy subjected to plastic working or obtained by casting, while the application of this technology to materials produced by additive methods remains an unexplored area of research. Based on the tests carried out (on samples before and after steam sterilization) and their comparison with mechanically polished samples without a diffusion layer, the following can be concluded:

- The most favorable results were observed for samples with a diffusion nitrogen layer from the HT_S_III group (with heat treatment at 910 °C and steam sterilization). These samples exhibited the highest resistance to pitting corrosion and the best resistance to tribological wear. The results obtained indicate that the presence of a nitride layer leads to increased resistance to abrasive wear, which extends the implant’s lifespan within the patient’s body. Furthermore, the reduction in abrasive wear minimizes the generation of metallic particles into the surrounding tissues, thereby reducing the risk of inflammatory reactions in the human body.
- A two-phase α + β structure was observed. In the case where microstructure of the material in its initial state, the α-phase was predominant. The heat-treated material at 800 °C comprised an α-phase matrix and an interlamellar β-phase. However, heat treatment at 910 °C and 1020 °C increased the proportion of the β-phase. Nevertheless, rapid cooling of the material after heat treatment led to the transformation of the β-phase into a metastable α’-phase.
- Based on the phase analysis, it can be concluded that the tested sample surfaces did not differ in phase composition or in peak intensities. This shows that the heat treatment of the alloy at 1020 °C had no effect on the structure of the layer. Based on the diffractograms obtained, it can be concluded that the formation of the diffusion layer resulted in the formation of TiN and Ti_2_N nitride layers. The resulting layer acts as a protective barrier between the substrate and the external environment. This is evidenced by the higher intensity of the peaks corresponding to the nitride phases compared to the peaks characteristic of the titanium substrate, which indicates the dominant contribution of the layer to the analyzed diffraction signal. Based on the conducted studies, changes can be observed in the pitting corrosion resistance tests, surface topography, and hardness. The authors also compare these results with those in the article without the deposited layer (after mechanical polishing) [13].
- The creation of a diffusion nitrogen layer significantly increased the Sa parameter value compared to the results of tests after mechanical polishing without a layer [13]. These values ranged from 0.52 μm to 0.56 μm. In the case of samples after steam sterilization, a decrease in this parameter was observed from 0.11 μm to 0.13 μm.
- The surfaces of samples after mechanical polishing [13] and with a diffusion nitrogen layer formed after steam sterilization are hydrophilic, which promotes cell proliferation processes on the surface [47,49].
- The presence of a diffusion nitrogen layer increased the hardness and abrasion resistance of all sample groups analyzed, indicating the beneficial effect of the nitriding process on the mechanical properties of the surface layer. Furthermore, the examination of tribological testing only provided a qualification rather than a quantitative assessment of abrasion resistance.
- The steam sterilization process can affect the physicochemical properties of the materials under investigation, leading to changes in their surface structure. The main cause of these changes is the phenomenon of oxidation, which occurs on the surfaces of metal alloys. Furthermore, the selection of steam sterilization parameters has a significant impact on the surface structure of the samples, leading to changes in the thickness of the resulting layers [1].

In further stages of research, it is planned to soak the samples in a PBS solution to simulate the physiological environment. Additionally, a layer degradation test will be carried out (an assessment of ions released into the solution).

## Figures and Tables

**Table 1:** Division of samples for testing.

Sample	Sample Group	Marking of Material for Testing *
As-built	I	AB_I
+heat treatment	II	HT_II
	III	HT_III
	IV	HT_IV
As-built after sterilization	I	AB_S_I
+heat treatment	II	HT_S_II
	III	HT_S_III
	IV	HT_S_IV

* A detailed division of the samples used in the study, along with the designation of individual groups, is presented in chapter 2.

**Table 2:** Technical and environmental parameters of the test.

Parameters of the Test	Unit	Pin
		Steel Ball—Sample
Load	N	1
Speed	cm/s	2
Number of cycles	-	1000
Frequency	Hz	0.5
Humidity	%	26
Temperature	°C	23 ± 1

**Table 3:** Microstructure of Ti6Al4V alloy.

Sample	Magnification	
	200×	1000×
**AB_I**	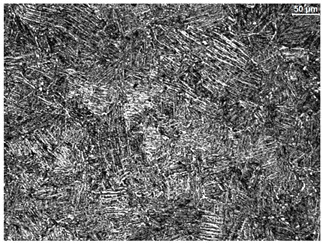	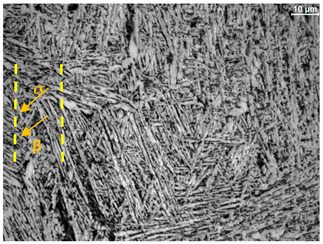
**HT_II**	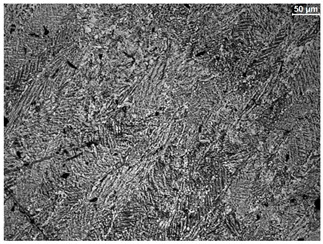	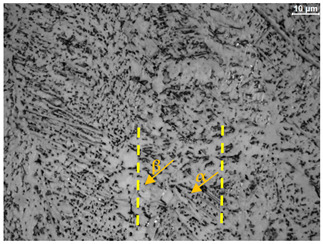
**HT_III**	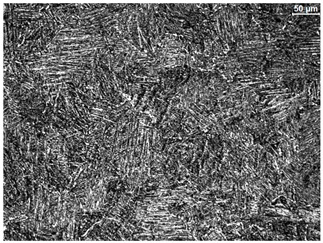	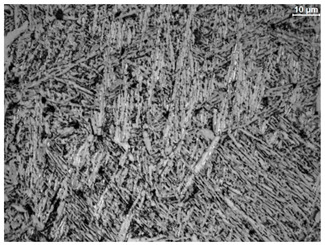
**HT_IV**	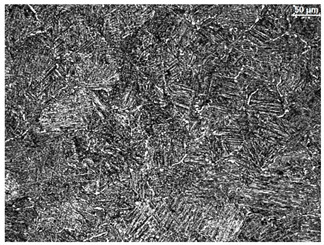	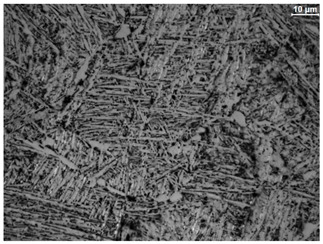

**Table 4:** Comparison of the Sa parameter for sample surfaces.

Sample	Sa [μm]	SD [μm]
**AB_I**	0.62	0.005
**HT_II**	0.52	0.011
**HT_III**	0.65	0.006
**HT_IV**	0.60	0.009
**AB_S_I**	0.13	0.006
**HT_S_II**	0.11	0.008
**HT_S_III**	0.12	0.003
**HT_S_IV**	0.14	0.002

SD—standard deviation.

**Table 5:** Pitting corrosion resistance results for samples before and after sterilization.

Sample	$E_{corr}$ [V]		$R_{p}$ $\left[kΩ\cdot {cm}^{2}\right]$		*E_np_* [V]		$E_{cp}$ [V]	
	Av.	SD	Av.	SD	Av.	SD	Av.	SD
**AB_I**	−0.187	0.005	83.2	2.9	3.4	0.1	1.3	0.8
**HT_II**	−0.101	0.005	72.9	1.2	3.8	0.2	1.2	1.3
**HT_III**	−0.012	0.008	76.2	6.9	3.7	0.2	2.0	0.1
**HT_IV**	−0.136	0.017	115.8	14.3	3.2	0.1	0.4	0.6
**AB_S_I**	−0.105	0.001	43.0	6.0	3.4	0.04	1.9	0.02
**HT_S_II**	−0.112	0.003	52.8	15.1	3.5	0.1	1.4	0.1
**HT_S_III**	−0.062	0.007	60.6	7.4	3.5	0.1	2.1	0.3
**HT_S_IV**	−0.119	0.067	39.0	3.8	3.5	0.2	0.8	0.1

**Av.**—*average*; **SD**—*standard deviation*.

**Table 6:** Surface of samples before and after corrosion resistance test.

Sample	Before Pitting Corrosion Test	After Pitting Corrosion Test
**AB_I**	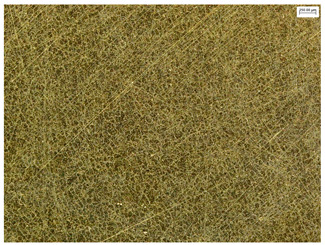	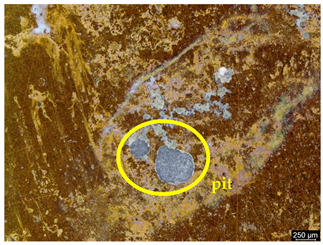
**AB_S_I**	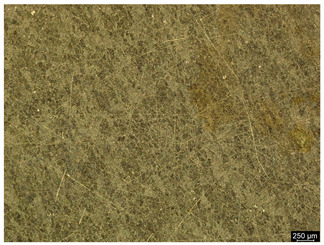	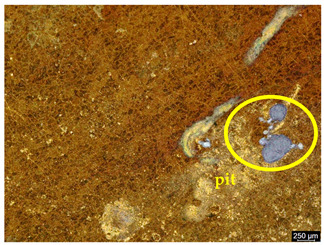
**HT_II**	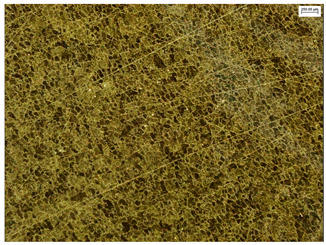	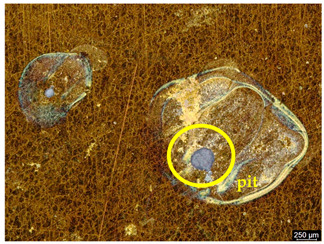
**HT_S_II**	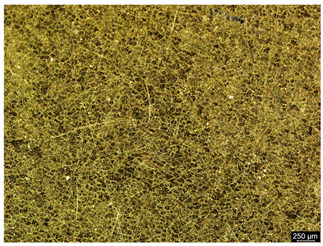	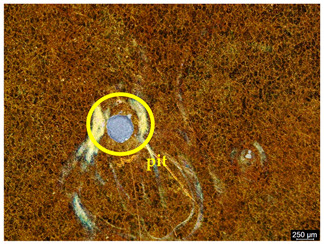
**HT_III**	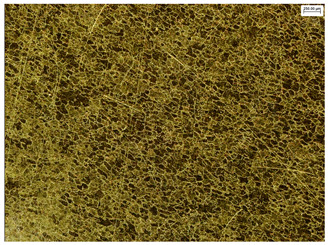	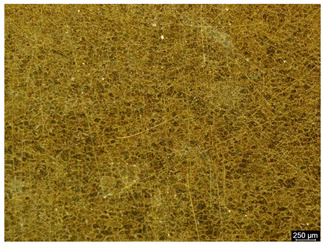
**HT_S_III**	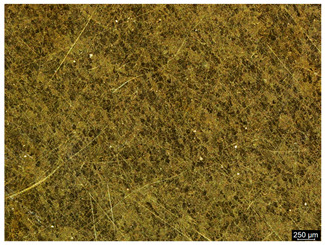	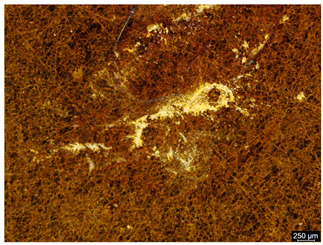
**HT_IV**	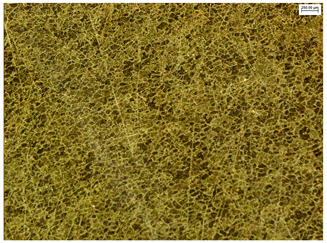	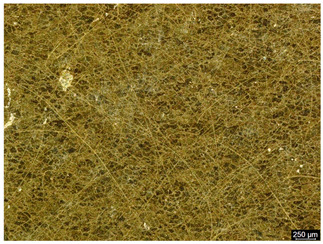
**HT_S_IV**	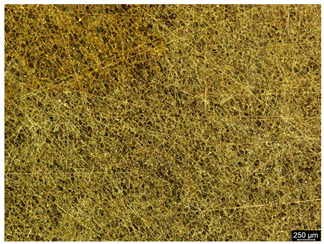	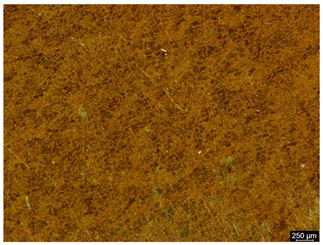

**Table 7:** Average friction coefficient.

Sample	Average Friction Coefficient
**AB_I**	1.00 ± 0.03
**HT_II**	0.97 ± 0.04
**HT_III**	0.99 ± 0.02
**HT_IV**	0.77 ± 0.11
**AB_S_I**	0.94 ± 0.08
**HT_S_II**	0.76 ± 0.25
**HT_S_III**	0.72 ± 0.04
**HT_S_IV**	1.14 ± 0.06

## Data Availability

The original contributions presented in this study are included in the article. Further inquiries can be directed to the corresponding author.
